# Associations between dietary patterns, *FTO* genotype and obesity in adults from seven European countries

**DOI:** 10.1007/s00394-022-02858-3

**Published:** 2022-03-21

**Authors:** Katherine M. Livingstone, Barbara Brayner, Carlos Celis-Morales, George Moschonis, Yannis Manios, Iwona Traczyk, Christian A. Drevon, Hannelore Daniel, Wim H. M. Saris, Julie A. Lovegrove, Mike Gibney, Eileen R. Gibney, Lorraine Brennan, J. Alfredo Martinez, John C. Mathers

**Affiliations:** 1 Institute for Physical Activity and Nutrition, School of Exercise and Nutrition Sciences, Deakin University, 1 Gheringhap St, Geelong, 3220 Australia; 2 BHF Glasgow Cardiovascular Research Centre, Institute of Cardiovascular and Medical Sciences, University of Glasgow, 126 University Pl, Glasgow, G12 8TA UK; 3 Human Performance Lab, Education, Physical Activity and Health Research Unit, University Católica del Maule, 3466706 Talca, Chile; 4 Department of Dietetics, Nutrition and Sport, School of Allied Health, Human Services and Sport, La Trobe University, Melbourne, VIC 3086 Australia; 5 Department of Human Nutrition, Faculty of Health Sciences, Medical University of Warsaw, Warsaw, Poland; 6 Department of Nutrition, Institute of Basic Medical Sciences, Faculty of Medicine, University of Oslo, Oslo, Norway; 7 Molecular Nutrition Unit, Department Food and Nutrition, Technische Universität München, München, Germany; 8 Department of Human Biology, NUTRIM, School for Nutrition and Translational Research in Metabolism, Maastricht University Medical Centre, Maastricht, The Netherlands; 9 Hugh Sinclair Unit of Human Nutrition and Institute for Cardiovascular and Metabolic Research, Department of Food and Nutritional Sciences, University of Reading, Reading, UK; 10 UCD Institute of Food and Health, UCD School of Agriculture and Food Science, University College Dublin, Belfield, Dublin 4 Republic of Ireland; 11 Precision Nutrition and Cardiometabolic Health, IMDEA-Food Institute (Madrid Institute for Advanced Studies), CEI UAM + CSIC, Madrid, Spain; 12 Human Nutrition Research Centre, Population Health Sciences Institute, Newcastle University, Newcastle upon Tyne, NE2 4HH UK

**Keywords:** Obesity, Waist circumference, Dietary patterns, *FTO* genotype, Adults

## Abstract

**Purpose:**

High-fat and low-fibre discretionary food intake and *FTO* genotype are each associated independently with higher risk of obesity. However, few studies have investigated links between obesity and dietary patterns based on discretionary food intake, and the interaction effect of *FTO* genotype are unknown. Thus, this study aimed to derive dietary patterns based on intake of discretionary foods, saturated fatty acids (SFA) and fibre, and examine cross-sectional associations with BMI and waist circumference (WC), and interaction effects of *FTO* genotype.

**Methods:**

Baseline data on 1280 adults from seven European countries were included (the Food4Me study). Dietary intake was estimated from a Food Frequency Questionnaire. Reduced rank regression was used to derive three dietary patterns using response variables of discretionary foods, SFA and fibre density. DNA was extracted from buccal swabs. Anthropometrics were self-measured. Linear regression analyses were used to examine associations between dietary patterns and BMI and WC, with an interaction for *FTO* genotype.

**Results:**

Dietary pattern 1 (positively correlated with discretionary foods and SFA, and inversely correlated with fibre) was associated with higher BMI (β:0.64; 95% CI 0.44, 0.84) and WC (β:1.58; 95% CI 1.08, 2.07). There was limited evidence dietary pattern 2 (positively correlated with discretionary foods and SFA) and dietary pattern 3 (positively correlated with SFA and fibre) were associated with anthropometrics. *FTO* risk genotype was associated with higher BMI and WC, with no evidence of a dietary interaction.

**Conclusions:**

Consuming a dietary pattern low in discretionary foods and high-SFA and low-fibre foods is likely to be important for maintaining a healthy weight, regardless of *FTO* predisposition to obesity.

**Trial registration:**

Clinicaltrials.gov NCT01530139. Registered 9 February 2012 https://clinicaltrials.gov/ct2/show/NCT01530139

**Supplementary Information:**

The online version contains supplementary material available at 10.1007/s00394-022-02858-3.

## Introduction

Globally, 52% of adults have overweight or obesity [1], and, in 2020, 65% of European adults were estimated to have overweight or obesity [2]. With these high prevalence rates for overweight and obesity, European adults are at increased risk of multiple chronic diseases and of higher all-cause mortality. Further, poor diet is a key modifiable risk factor for chronic disease [3]. In particular, diets high in discretionary foods and beverages that are high in saturated fatty acids (SFA), and low in fibre, are linked with greater risk of obesity, as well as cardiovascular disease and all-cause mortality [4–8].

Since foods and nutrients are not consumed in isolation, research considering the frequency and combinations of foods consumed as part of an overall dietary pattern is gaining importance [9]. Methods used to describe dietary patterns, such as reduced rank regression, combine the strengths of *a priori* knowledge of diet–health associations and data driven methods [10, 11]. Although dietary guidelines recommend consuming a dietary pattern low in discretionary foods and high in fibre-rich foods [12], no studies have derived dietary patterns based on percentage energy intake from discretionary foods, SFA and intake of fibre.

There is consistent evidence that unhealthy dietary patterns are associated with increased risk of obesity [13]. Moreover, associations between non-modifiable risk factors, such as genetic variations in the fat mass and obesity-associated gene (*FTO*), and obesity, may be modified by diet [13–15]. The *FTO* gene is postulated to regulate energy homeostasis [16] and the risk genotype has been associated with altered macronutrient intakes, such as total fat [17], and higher odds of consuming a dietary pattern high in discretionary foods [18]. Thus, in individuals with the risk genotype, maintaining a healthy dietary pattern may be more important for reducing risk of obesity. In a nested case–control study of 1 254 adults, higher adherence to the Mediterranean diet was associated with lower obesity risk among subjects with higher genetic predisposition to obesity, when compared with those with lower adherence and lower genetic risk. [14]. However, previous cross-sectional research in the Food4Me study has shown no evidence of interaction between a Mediterranean diet score and *FTO* genotype on anthropometrics [47].

Discretionary food intake and *FTO* genotype are each associated independently with higher risk of obesity [16, 19]. However, while studies have investigated links between obesity and dietary patterns high in discretionary foods, such as processed meat [11, 20, 21], no studies have used *a posteriori* methods that derive dietary patterns based on discretionary food intake. Moreover, the association between *FTO* genotype and such dietary patterns, as well as the interaction effect between these dietary patterns and *FTO* genotype on associations with obesity is unknown. As dietary guidelines are increasingly focused on dietary patterns, rather than single foods, understanding how high-SFA and low-fibre discretionary foods are eaten in combination will inform the design of dietary pattern-based recommendations for the prevention of obesity. Therefore, the objective of this study was to derive dietary patterns based on intake of discretionary foods and beverages, SFA and fibre, and to examine cross-sectional associations with obesity and moderating effects of *FTO* genotype.

## Methods

### Study design

This study was a cross-sectional analysis of baseline data from the Food4Me study, a 6-month, randomised controlled trial in seven European countries (United Kingdom; Ireland; Spain; Greece; The Netherlands, Germany, Poland) [22]. Briefly, individuals were recruited via the Food4Me website following flyers, newspaper and radio advertisements [22]. Participants were asked to complete an online questionnaire via e-mail and to provide biological samples at baseline and after 3 and 6 months of intervention. Participants completed an online food frequency questionnaire (FFQ), the Baecke physical activity questionnaire [23], wore accelerometers, and provided self-measured anthropometric information, buccal swabs and dry blood spot cards. Recruitment was targeted at 1540 participants (i.e., *n* = 220 participants per country) and was conducted between August 2012 and August 2013. Each research centre or university supervising the intervention obtained Research Ethics Committee approval for the study from their local or national committee. The Food4Me study was registered at www.clinicaltrials.gov under the number NCT01530139. Online consent forms were signed by participants. Reporting was guided by the STROBE-nut guidelines for nutritional epidemiology (Supplementary Table 1).

### Eligibility criteria

Participants aged 18 years and older were included in the Food4Me study. The following exclusion criteria were employed: (I) pregnant or lactating; (II) no or limited access to the Internet; (III) following a prescribed diet for any reason, including weight loss in the last 3 months; (IV) diabetes, coeliac disease, Crohn’s disease or any metabolic condition altering nutritional requirements. For this cross-sectional analysis, participants were included if they had complete data for exposures, outcomes, confounders and moderators.

### Study variables

#### Dietary intake

An online semi-quantitative FFQ was used to estimate dietary intake. The FFQ included 157 food and beverages consumed frequently in each of the participating European countries and had been developed and validated specifically for the Food4Me study [24, 25]. Detailed information about the development, reproducibility, and validity of the FFQ are provided elsewhere [24, 26]. Food and nutrient composition were computed in real time using the *McCance and Widdowson’s The composition of Foods* food composition database [27].

The percentage energy (%E) from discretionary food and beverage intakes was calculated based on foods and beverages included in the Food Standards Scotland classification of discretionary items [28]. A total of 22 items from the Food4Me study FFQ were included in this discretionary food classification from the following food groups: cakes, pastries and puddings (8 items), crisp and savoury snacks (4 items), confectionary (3 items), sugar containing drinks (3 items), ice cream and desserts (2 items) and sweet biscuits (2 items). A full list of the foods and beverages included in the discretionary classification used in the present study is provided in Supplementary Table 2 and published previously [29]. Reported mean daily intake of discretionary food items were summed and the energy intake from discretionary items was divided by the total mean daily energy intake and multiplied by 100.

#### Dietary patterns

Dietary pattern scores were derived in SAS using reduced rank regression, using food and nutrient intake data collected from the FFQ. Reduced rank regression constructs dietary pattern scores by creating linear combinations (factor scores) of food‐groups that maximize the explained variation in the response variables (i.e., nutrient intakes) that are hypothesized to be related to health outcomes [30]. In this study, the predictor variables were 45 food groups (Supplementary Table 3). These food groups were created based on comparable nutrient composition and according to the groupings used in the UK National Diet and Nutrition Survey [31]. This national survey was selected as it was European, and provided comparability with the UK-based discretionary foods classification [32]. The number of dietary patterns extracted depends on the number of nutrient response variables. Thus, in this analysis, three nutrients known to be associated with obesity risk were used as response variables: %E from SFA, fibre density (g/MJ) and %E from discretionary foods. These nutrients were selected based on evidence from the World Health Organisation’s report on chronic disease prevention that fibre density and saturated fat intake are strongly associated with obesity risk [33], and national guidelines to reduce intake of discretionary foods [12]. Based on our previous applications of reduced rank regression, we will explore all dietary patterns created that account for > 10% variation [11, 20] and are interpretable based on their nutrient and food group intakes.

#### Overweight and obesity and central obesity

Participants were provided with online information sheets and online video instructions on how to complete anthropometric measurements. Weight (kg) and standing height (cm) were self-reported. Body Mass Index (kg/m^2^) was calculated using the standard formula of weight (kg) divided by height (m^2^). Participants self-collected waist circumference using a tape measure, measured between the lowest rib margin and iliac crest, horizontally when standing up straight. Self-reported measurements were validated in a sub-sample of the participants (*n* = 140) and showed a high degree of reliability [34]. Overweight and obesity status (binary) was assessed by creating an underweight/normal weight (BMI < 25 kg/m^2^) category and an overweight or obesity category (BMI ≥ 25 kg/m^2^) [35]. Central obesity was defined as a waist circumference (WC) ≥ than 102 cm for men and ≥ than 88 cm for women [36].

#### Genotyping

Buccal cell samples were collected by participants using Isohelix SK-1 DNA buccal swabs and Isohelix dried capsules and posted to each recruiting centre. The recruitment centres shipped these samples to LCG Genomics, UK, which then extracted the DNA and genotyped specific loci using KASP™ genotyping assay to provide bi-allelic scoring of *FTO* SNP *rs9939609* and *rs1121980*. Since there is high linkage disequilibrium (*r* ^2^ 0.96) between these two SNPs, the results for *rs1121980* are not reported. No significant deviation from the Hardy–Weinberg equilibrium was observed for *rs9939609* (0.51; *P* = 0.48). The additive model of *FTO rs9939609* was used (TT, AT, AA).

#### Demographic and lifestyle information

An online questionnaire collected information on age, sex, country, occupation, physical activity and smoking status. Based on European classifications of occupations the following groupings were used: ‘Professional’ (professionals; managers); ‘Intermediate’ (armed forces occupations; technicians and associate professionals; clerical support workers); and ‘Manual’ (craft and related trades workers; plant and machine operators and assemblers; service and sales workers; elementary occupations; skilled agricultural, forestry and fishery workers) [37]. Categories for ‘Students’ and ‘Retired and unemployed’ were added. Physical activity level was estimated from triaxial accelerometers (TracmorD, Philips Consumer Lifestyle), and identified minutes per day spent in physical activity. A binary variable was created for descriptive purposes to reflect whether participants met physical activity recommendations (> 150 min of moderate physical activity or > 75 min vigorous physical activity or a combination of moderate and vigorous physical activity in a week in bouts of at least 10 min) [38]. Smoking was defined as current smoker or ex/non-smoker. As per previous use of these data, analyses were also adjusted for energy misreporting (yes/no) [29]. Under reporters were individuals with reported energy intake lower than basal metabolic rate *1.1. Basal metabolic rate was calculated based on Oxford equations [39]. Over reporters were individuals with reported energy intake higher than 4500 kcal per day [40].

### Statistical analysis

Complete case analysis was used. Descriptive statistics were used for participant characteristics and are presented as means and standard deviation for continuous variables or frequency counts for categorical variables. Unadjusted linear regression analyses were used to examine associations between dietary patterns and total energy (MJ/day) and nutrient intake (%E from carbohydrate, total sugars, protein, total fat, trans fat, poly-unsaturated fat, mono-unsaturated fat, alcohol and omega-3 [g/day] and fibre [g/day]). Logistic and linear regression analyses were used to examine associations between dietary patterns (dependent variable) and obesity and central obesity (independent binary variables) as well as between dietary patterns and BMI and waist circumference (independent continuous variables). Dietary patterns were treated as categorical variables (tertiles of dietary pattern score) and continuous variables; tertiles were used to help describe the food group and nutrient intakes according to these dietary patterns, while continuous variables were used to maximise the statistical power of the regression analyses. The outcomes were risk of overweight/obesity (binary), central obesity (binary), BMI (continuous) and WC (continuous). Regression analyses were adjusted for the following confounders based on previous literature: age (continuous), sex (categorical), country (categorical; United Kingdom; Ireland; Spain; Greece; The Netherlands, Germany, Poland), physical activity (continuous), smoking status (binary) and energy misreporting (binary). Margins plots were created for each dietary pattern and continuous outcomes (BMI and WC). An interaction effect of *FTO* genotype (categorical) on associations between dietary patterns and obesity status, central obesity, BMI and WC was tested by including a multiplicative interaction term between *FTO* and dietary patterns in the model. Linear regression analyses were also used to examine the association between dietary patterns (continuous) and *FTO* genotype (categorical), with and without adjustment for BMI (continuous). To further investigate the effects of energy misreporting, analyses were repeated excluding individuals who were considered under or over reporters. Where the interaction was not significant, main effects without the interaction terms were presented. Dietary patterns were generated in SAS on demand software, whereas all other analyses were performed in Stata SE 15 (64 bit). All results were considered significant when *p* value < 0.05.

## Results

Of the 1607 participants in Food4Me randomised into the intervention at baseline, 327 participants had missing data for the exposure, confounders and/or moderator (20%), thus 1,280 participants were included in the present analysis. Participant characteristics of those who were included in this analysis compared to those who were excluded were on average more likely to be female, younger and adults with obesity than those who were excluded [41]. Participants included in this study had a mean age of 40.4 (SD 13.0) years, 58% were women, 11% were smokers and 77% met physical activity recommendations. Forty six percent of participants had a BMI ≥ 25 kg/m^2^ (Table 1). Participant characteristics by weight status are presented in Supplemental Table 4. Overall, the %E from discretionary foods was 13.6 (SD 9.5) % and from SFA was 14.1 (SD 3.6) % and fibre density was 2.8 (SD 0.9) g/MJ. Twenty-one percent of participants were energy intake misreporters (16% under-reported and 5% over-reported energy intake; data not shown).

**Table 1:** Participant characteristics overall and by tertile of dietary patterns (*n* = 1 280)^1^

Characteristics	Overall	Dietary pattern 1^2^			Dietary pattern 2^2^			Dietary pattern 3^2^		
		Tertile 1	Tertile 2	Tertile 3	Tertile 1	Tertile 2	Tertile 3	Tertile 1	Tertile 2	Tertile 3
Age, years	40.4 ± 13.0	42.6 ± 13.9	39.7 ± 12.8	38.9 ± 12.0*	40.9 ± 12.6	39.8 ± 13.4	40.5 ± 13.0	39.6 ± 13.2	39.5 ± 12.7	42.1 ± 13.0*
Female (%)	58.1	58.8	58.3	57.0	50.8	59.5	63.8*	41.5	62.8	70.0*
Country (%)										
Germany	13.6	10.3	13.4	17.1*	17.8	13.8	9.2*	7.5	12.9	20.4*
Greece	13.6	10.8	17.1	12.9	13.4	17.6	9.7	15.0	15.9	10.0
Ireland	14.0	13.4	15.5	13.2	11.0	12.4	18.5	10.3	14.7	16.9
Netherlands	16.7	25.3	13.8	11.0	12.7	15.2	22.3	14.5	17.1	18.5
Poland	13.9	13.4	12.9	15.3	18.7	13.4	9.4	11.2	12.9	17.4
Spain	14.1	14.5	13.4	14.6	15.2	14.1	13.2	26.7	11.7	4.0
United Kingdom	14.1	12.4	14.1	16.0	11.2	13.6	17.6	14.7	14.7	12.9
Occupation (%)										
Professional	40.2	40.5	40.3	39.7*	41.9	36.8	41.8*	37.9	43.8	38.7*
Intermediate	24.9	22.3	23.7	28.9	27.4	24.3	23.0	25.5	23.7	25.6
Manual	10.1	8.0	11.2	11.0	11.7	10.7	8.5	12.7	8.2	9.4
Student	14.4	15.5	14.8	12.9	11.2	15.7	16.2	16.2	13.6	13.4
Retired/unemployed	11.5	13.8	10.1	7.5	7.7	13.1	10.6	7.7	10.8	12.9
Smoker (%)	11.4	7.8	11.9	14.5*	14.3	11.5	8.5*	15.2	9.6	9.4*
Meet PA recommendations (%)	77.3	80.1	77.7	74.2	76.4	76.6	79.1	77.7	78.2	76.1
*FTO* rs99397609 (%)										
TT	31.6	28.8	35.8	30.3	31.1	31.2	32.6	33.5	30.2	31.2
TA	50.2	53.4	48.0	49.0	50.6	50.8	49.1	49.2	49.4	51.9
AA (risk variant)	18.2	17.8	16.2	20.7	18.3	18.0	18.3	17.3	20.4	16.9

*SD* standard deviation, *PA* physical activity

1, Values mean ± SD for age; PA recommendations: > 150 min of moderate physical activity or > 75 min vigorous physical activity or a combination of moderate and vigorous physical activity in a week in bouts of at least 10 min

2, Unadjusted linear regression analyses were used to examine P trend across tertiles of dietary pattern for age; Chi squared were used to test for significant differences across other variables. Significance at *P* < 0.05 are indicated *. Tertile 1: *n* = 427; Tertile 2: *n* = 427; Tertile 3: *n* = 426

### Dietary patterns

Three dietary patterns were created, designated DP1, DP2 and DP3, explaining a total of 46.6%, 18.7% and 11.7% of variation in the response variables, respectively (Table 2). DP1 was moderately positively correlated with %E from SFA (*r* 0.59) and discretionary foods (*r* 0.52) and moderately negatively correlated with fibre density (*r* − 0.62) suggesting participants with higher DP1 scores were consuming more foods high in SFA and discretionary foods, while having a lower intake of fibre dense foods. DP1 was positively associated with intake of sweet biscuits and confectionary and butter and negatively associated with high fibre breakfast cereals and wholemeal pasta and rice (Table 3). Participants in the highest tertile of DP1 had higher intake of total fat and lower intake of fibre as compared with participants in the lowest tertile (Supplementary Table 5). DP2 was strongly positively associated with %E from discretionary foods (*r* 0.86), weakly positively associated with fibre density *(r* 0.39) and weakly negatively correlated with %E from SFA (*r* − 0.33) intake, suggesting participants with higher DP2 scores consumed higher amounts of discretionary foods and lower amounts of foods high in SFA. DP2 was associated with higher intake of confectionary and sweet biscuits and lower consumption of beef and veal and butter (Table 3). Participants in the highest tertile of DP2 had higher intakes of carbohydrate, fibre and total sugar, but lower intakes of protein and total fat when compared with participants in the lowest tertile (Supplementary Table 5). DP3 was strongly positively associated with %E from SFA *(r* 0.73), moderately positively correlated with fibre density (*r* 0.68) and was weakly negatively correlated with %E from discretionary foods (*r* − 0.03), suggesting that participants with higher DP3 scores consumed higher amounts of SFA and fibre dense food. DP3 was associated with higher butter and vegetables intake and lower consumption of spirits and white bread (Table 3). Participants in the highest tertile of DP3 had higher intakes of total fat and fibre when compared with those in the lowest tertile (Supplementary Table 5). A full list of factor loadings for all dietary patterns is presented in Supplementary Table 6.

**Table 2:** Explained variation (%) in food intakes and nutrient response variables for each dietary pattern, and correlation coefficient between dietary patterns and response variables (*n* = 1280)

DP	Explained variation (%)^1^					Correlation coefficient		
	Total		Nutrient response variables					
	Food group intakes	Nutrient response variables	SFA	Fibre density	Discretionary foods	SFA	Fibre density	Discretionary foods
DP1	4.6	46.6	48.8	53.6	37.3	0.59	− 0.62	0.52
DP2	2.7	18.7	55.2	62.3	78.5	− 0.33	0.39	0.86
DP3	3.2	11.7	74.1	78.5	78.5	0.73	0.68	− 0.03

*DP* Dietary pattern, *SFA* saturated fatty acids, *%E* percentage from total energy intake

^1^Explained variation (*R* square) represents the proportion of the variance accounted for by RRR factors. For example, for the nutrient response variables, this is the amount of the dependent variable (response variables, i.e., SFA, fibre density and discretionary foods) that is predictable from the independent variable (food groups)

**Table 3:** Intake of nutrients used as response variables in the derivation of the dietary patterns and top five positive and negative loading food groups across tertiles of dietary patterns (*n* = 1280) P for trend from unadjusted linear regression analysis across tertiles of dietary pattern; all < 0.001. Tertile 1: *n* = 427; Tertile 2: *n* = 427; Tertile 3: *n* = 426

Food groups	Factor loading	Tertiles of dietary pattern					
		Tertile 1		Tertile 2		Tertile 3	
		Mean	SD	Mean	SD	Mean	SD
Dietary pattern 1							
Response variables							
SFA (%E)	**–**	11.7	2.32	14.0	2.13	16.7	2.66
Fibre density (g/MJ)	**–**	3.70	0.86	2.70	0.56	2.08	0.56
Discretionary (%E)	**–**	8.19	5.13	12.7	6.68	20.0	11.4
Positive associations (g/d)							
Sweet biscuits	0.34	12.9	21.4	21.0	36.3	49.4	92.6
Confectionary	0.33	10.8	12.8	14.8	15.9	33.2	43.5
Cakes, pastries and pudding	0.25	27.5	26.5	31.2	24.5	58.8	64.7
Butter	0.25	3.38	7.78	4.68	8.88	10	18.4
Pizza and snacks	0.21	24.3	24.4	32.0	26.2	49.6	54.2
Negative associations (g/d)							
High fibre cereals	− 0.16	85.6	118	44.2	64.7	31.8	52.7
Whole meal pasta and rice	− 0.17	36.6	58.4	16.2	26.7	11.6	23.7
Whole meal breads	− 0.21	148	167	70.7	85.7	64.0	100
Vegetables	− 0.23	208	129	147	88	123	78.1
Fruits	− 0.31	421	311	229	162	178	143
Dietary pattern 2							
Response variables							
SFA (%E)	–	15.6	3.09	13.6	2.65	13.2	3.09
Fibre density (g/MJ)	–	2.39	0.67	2.88	0.81	3.22	1.08
Discretionary (%E)	–	8.49	4.99	12.3	6.38	20.1	11.7
Positive associations (g/d)							
Confectionary	0.39	12.4	14.7	15.0	16.0	31.3	43.7
Sweet biscuits	0.34	12.4	22.5	16.9	24.1	54	94.6
Cakes, pastries and pudding	0.25	30.7	29.8	33.7	28.7	53.1	63.7
Fruits	0.25	205	171	253	195	371	308
Crisps and savory snacks	0.17	3.08	6.69	3.29	6.03	5.50	10.2
Negative associations (g/d)							
Beef and veal	− 0.16	61.0	76.8	43.1	35.7	42.1	42.0
Butter	− 0.17	9.96	16.2	4.59	9.12	3.46	11.4
Eggs and egg dishes	− 0.18	41.8	52.2	26.6	30.5	24.2	30.7
Cheese	− 0.20	27.2	29.7	17.0	18.4	15.2	18.0
Whole milk	− 0.23	313	368	169	206	130	205
Dietary pattern 3							
Response variables							
SFA (%E)	–	12.8	2.43	13.9	2.70	15.6	3.51
Fibre density (g/MJ)	–	2.35	0.64	2.89	0.78	3.25	1.09
Discretionary (%E)	–	14.0	9.57	13.8	9.22	13.1	9.84
Positive associations (g/d)							
Butter	0.37	2.17	6.30	3.79	6.94	12.1	18.9
Vegetables	0.30	124	75.6	139	76.2	214	135
Fruit	0.29	193	166	248	169	387	317
Cheese	0.23	14.1	18.1	17.4	18.3	27.9	29.4
Tea and Coffee	0.17	351	404	428	442	582	527
Negative associations (g/d)							
Spirits and other	− 0.15	7.13	14.86	4.17	8.04	2.98	7.29
White bread	− 0.20	266	348	155	128	186	163
Sugar containing soft drinks	− 0.20	72.2	213	33.9	73.4	26.3	58.9
Non-fried poultry	− 0.25	57.7	43.4	35.3	30.9	31.8	36.9
Beer and cider	− 0.28	162	236	65.6	106	45.4	94.7

*SFA* saturated fatty acids, *%E* percentage from total energy intake

^1^

### Dietary patterns and participant characteristics

More individuals in the highest tertile of DP1 were younger, smokers, in intermediate and manual occupations and from Germany. More individual in the highest tertile of DP2 were female, non-smokers, students, and from the Netherlands and the United Kingdom. More individuals in the highest tertile of DP3 were older, female, non-smokers, retired or unemployed, and from Germany, Ireland and Poland (Table 1).

### Dietary patterns and overweight/obesity

**Fig. 1:**
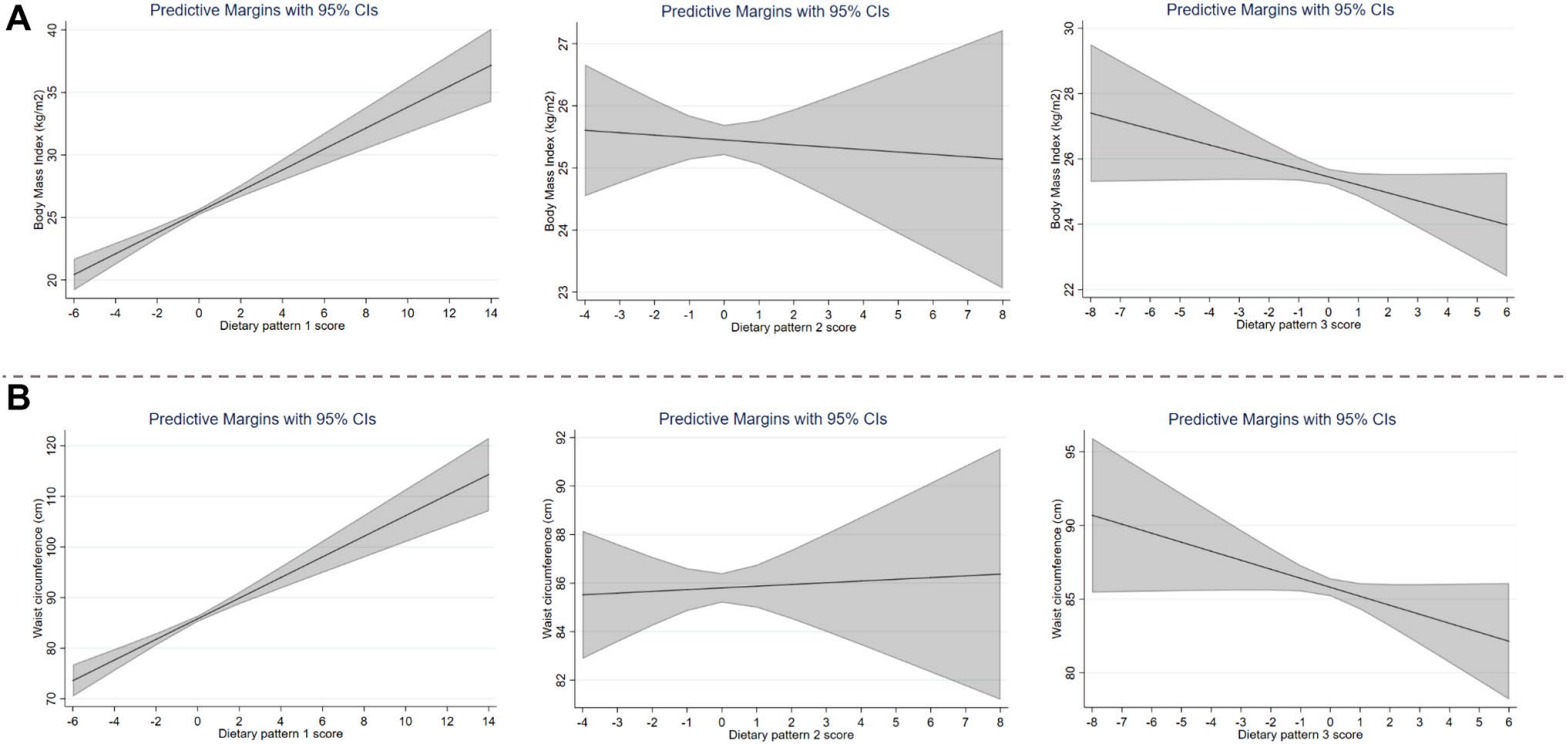
Association of the dietary pattern scores with body mass index (**A**) and waist circumference (**B**). Data are presented as adjusted means and their 95% CI; adjusted for age, sex, smoking status, country, physical activity, and energy misreporting

As shown in Table 4, individuals in the highest tertile of DP1 (positively correlated with SFA and discretionary foods, and negatively with fibre density), had higher odds of having overweight/obesity (OR: 2.39; 95% CI 1.75, 3.27). These associations remained consistent when DP1 was treated as a continuous variable (OR: 1.38; 95% CI 1.23, 1.55), and when BMI was treated as a continuous variable (beta coefficient for BMI per DP unit increase: 0.64; 95% CI 0.44, 0.84) (Fig. 1; Supplementary Table 7). Individuals in the highest tertile of DP1 also had higher odds of having central obesity (OR: 4.27; 95% CI 2.77, 5.56), with results remaining consistent when DP1 was treated as a continuous outcome (OR: 1.32; 95% CI 1.16, 1.49), and when WC was treated as a continuous variable (beta coefficient for WC per dietary pattern unit increase: 1.58; 95% CI 1.08, 2.08). There was some evidence that DP2 with inversely associated with risk of overweight/obesity (Table 4). There was some evidence that DP3 was inversely associated with WC when treated as a continuous variable (beta coefficient for WC per dietary pattern unit increase: − 0.67; 95% CI − 1.32, − 0.03; Fig. 1; Supplementary Table 7). After excluding energy misreporters, results remained consistent for DP1 and all dietary patterns became significantly associated with WC (Supplementary Table 8).

**Table 4:** Associations between dietary patterns and anthropometrics, with interaction effects by *FTO* genotype (*n* = 1280)

	Dietary pattern (continuous)				Dietary pattern (tertiles)						
	Odds ratio^a^	95% CI	*P* value^a^	P_interaction_^c^	Tertile 1 (ref)	Tertile 2		Tertile 3		P_trend_^b^	P_interaction_^c^
					Odds ratio	Odds ratio	95% CI	Odds ratio	95% CI		
Overweight/obesity^d^											
Dietary pattern 1	1.38	1.23, 1.55	< 0.001	0.47	1.00	1.67	1.23, 2.28	2.39	1.75, 3.27	< 0.001	0.96
Dietary pattern 2	0.93	0.82, 1.06	0.28	0.84	1.00	0.79	0.59, 1.07	0.71	0.52, 0.97	0.028	0.95
Dietary pattern 3	0.90	0.79, 1.03	0.12	0.64	1.00	0.91	0.67, 1.23	0.88	0.64, 1.22	0.44	0.92
At risk WC^d^											
Dietary pattern 1	1.32	1.16, 1.49	< 0.001	0.10	1.00	1.58	1.10, 2.27	1.96	1.10, 2.27	< 0.001	0.68
Dietary pattern 2	1.01	0.88, 1.17	0.89	0.40	1.00	0.77	0.54, 1.08	0.84	0.59, 1.19	0.31	0.21
Dietary pattern 3	0.89	0.77, 1.04	0.14	0.45	1.00	0.81	0.57, 1.16	0.78	0.54, 1.14	0.20	0.99

*T* tertile, *WC* waist circumference

^a^For a one-unit increase in the dietary pattern predictor variable, the odds of overweight/obesity and “at risk” WC (the dependent variables) being positive (= 1) increases by factor ‘X’, holding age, sex, smoking status, country, physical activity, and energy misreporting constant

^b^P for trend across tertiles of dietary patterns. Logistic regression analyses were adjusted for age, sex, smoking status, country, physical activity, and energy misreporting

^c^P for interaction of *FTO* rs99397609 genotype (categorical) on the association between dietary pattern (continuous and categorical) and anthropometrics. Logistic regression analyses were adjusted for age, sex, smoking status, country, physical activity, and energy misreporting

^d^Underweight/normal weight: body mass index < 25 kg/m^2^, Overweight/obesity: body mass index ≥ 25 kg/m^2^

^e^At risk WC: WC ≥ 102 cm for men and ≥ 88 cm for women

### *FTO* genotype

Overall, 18% of participants were homozygous for the *FTO* risk genotype (Table 1). The proportion of individuals homozygous for the *FTO* risk genotype was higher in individuals with obesity (20.9%) compared with those without obesity (15.9%) (Supplementary Table 4). There was no evidence that dietary patterns were associated with *FTO* risk genotype (DP1: *P* = 0.77; DP2: *P* = 0.66; DP3: *P* = 0.58). There was limited evidence of an interaction effect between DP1 and *FTO* risk genotype on obesity outcomes and no evidence for DP2 and DP3 (Table 4).

## Discussion

This large pan-European study identified a dietary pattern high in percentage energy from SFA and discretionary foods and beverages and low in fibre density that was associated with higher BMI and WC. Although carrying the *FTO* risk genotype was also associated with higher BMI and WC, no interaction was observed between this dietary pattern and the *FTO* genotype. These findings highlight the importance of limiting intake of high SFA and low fibre discretionary foods for maintaining a healthy weight, regardless of genetic predisposition to obesity from the *FTO* genotype.

The dietary pattern characterised by high intake of discretionary food and beverages identified in this study is comparable with energy-dense dietary patterns identified in previous studies [11, 42]. While no studies in adults have used reduced rank regression with these response variables, a prospective study of 521 children living in the UK identified an energy dense and low fibre dietary pattern high in crisps and snacks, chocolate, and confectionery that was associated with higher fat mass and greater odds of excess adiposity in childhood [42]. Our present study is the first to derive dietary patterns based on the Food Standards Scotland definition of discretionary foods, which does not include processed meat and most alcoholic beverages. Thus, in contrast with our study, most energy-dense dietary patterns identified in adult populations typically include processed meats and alcohol [43, 44]. For example, in a cross-sectional study of 2 197 UK adults, cluster analyses of weighed dietary records identified a prevalent dietary pattern in men as “beer and convenience foods,” which included high intakes of processed meat, chips and beer [44]. This dietary pattern was often consumed by smokers and participants in manual occupations as was dietary pattern 1 in our present study. Although some high sugar alcoholic beverages were included in the response variable used to derive the discretionary dietary pattern (DP1) in our study, as expected, alcoholic beverages loaded low in the resulting pattern.

Consistent with our findings, previous research has shown positive associations between a high discretionary food dietary pattern and BMI and WC [45, 46]. In a cross-sectional nationally representative sample of 4,908 Australian adults, a high-fat and low fibre dietary pattern, derived using reduced rank regression, was positively associated with prevalence of overall and central obesity [11]. Furthermore, a study in a cross-sectional and nationally representative sample of 9,688 US adults showed that dietary energy density was positively associated with BMI and WC [46]. However, some energy-dense foods are also nutrient-dense (e. g. cheese and nuts), and are thus not synonymous with discretionary foods. As relatively few studies have derived dietary patterns using reduced rank regression, and no studies have used percent energy from discretionary foods and beverages as a response variable, direct comparisons with previous studies are not possible. Nonetheless, prospective research in children suggests that diets characterised by energy-dense foods high in fat and low in fibre are associated with higher odds of obesity [42]. Further prospective studies in adults are needed to confirm whether this discretionary food dietary pattern contributes towards the development of obesity.

Evidence for an interaction effect of dietary patterns and *FTO* genotype on obesity risk remains inconclusive [47]. Previous cross-sectional analysis in this cohort has shown some evidence of an interaction between sugar-sweetened beverage intake and *FTO* genotype, but there was no evidence of interactions with nutrient intakes and overall diet quality, as estimated using the HEI-2010 and Mediterranean Diet Score [47]. In contrast, a cross-sectional and case–control study in Middle Eastern adults and the PREDIMED randomised controlled trial have shown some evidence of diet–gene interactions for specific nutrients, such as fibre and total fat, and the Mediterranean diet [48–50]. However, these studies included either non-Caucasian populations or participants at high risk of cardiovascular disease, which limits comparability with our present study. Moreover, since more than 100 genetic variants are associated with obesity [51], further research on this topic should use polygenic risk scores, based on multiple SNPs, to define genetic predisposition to obesity.

Several limitations should be acknowledged. All data collected during the study were self-reported with the potential for measurement errors, such as misreporting of energy intake. Nonetheless, protocols were standardised across all centres, delivered in the language of each country and participants were assisted in their recording of information, and in sample collection, by the provision of detailed instructions, video clips and frequently asked questions. Furthermore, an embedded validation study in which anthropometric measurements were replicated by a trained researcher showed good agreement with self-reported values [34] and the FFQ has been validated against 4-day weighed food records [25]. Reverse causality cannot be discounted due to the cross-sectional study design and, although we adjusted for key confounders known to impact our associations, residual confounding may remain. Regarding the dietary patterns, although the food groups used were based on the UK National Diet and Nutrition Survey and the response variables were selected based on published literature, the number and choice of food groups and response variables are somewhat subjective and may have influenced the derived dietary patterns. Finally, the diet–gene interactions, particularly between DP1 and *FTO* on WC, require testing in a larger cohort to determine whether results were due to the study being insufficiently powered to detect interactions. The present study had a number of strengths. The Food4Me study included participants from seven European countries. Although the sample was self-selected adults who may be more health-conscious than the general population, participants had similar lifestyle behaviours to the European adult population with potential to benefit from improvements in diet and lifestyle behaviours. A further strength of our study was the use of reduced rank regression, which utilised both prior knowledge of nutrients known to be associated with obesity risk and data-driven dietary pattern methods.

In conclusion, this pan-European study identified a dietary pattern high in percentage energy from SFA and discretionary foods and beverages, and low in fibre density that was associated with higher odds of overall and central obesity. While the *FTO* risk genotype was associated with higher odds of overall and central obesity, there was no interaction effect between this dietary pattern and *FTO* genotype. These findings have the potential to inform food- and nutrient-based dietary guidelines for obesity prevention, because our results reinforce recommendations to increase consumption of foods high in fibre, such as fruit, vegetables and wholegrains, while minimising intake of discretionary foods high in SFA and low in fibre. Future research should confirm these findings by examining prospective associations with obesity risk.

## Supplementary Information

Below is the link to the electronic supplementary material.

Supplementary file1 (DOCX 61 KB)

## Data Availability

The data sets used and/or analysed during the current study are available from the corresponding author on reasonable request.

## Abbreviations

BMI: Body mass index
DP: Dietary pattern
FTO: Fat mass and obesity associated
FFQ: Food frequency questionnaire
RCT: Randomised controlled trial
SFA: Saturated fatty acid
WC: Waist circumference
