# Supplementary material for: Associations between dietary patterns, FTO genotype and obesity in adults from seven European countries
Source: Eur J Nutr. 2022 Mar 21;61(6):2953–65. doi: 10.1007/s00394-022-02858-3 (PMC9363276; doi:10.1007/s00394-022-02858-3)
Supplement: Supplementary file 1 — Supplementary file1 (DOCX 61 KB) [file 394_2022_2858_MOESM1_ESM.docx]

Supplementary Table 1 STROBE-nut: An extension of the STROBE statement for nutritional epidemiology

| **Item** | **Item nr** | **STROBE recommendations** | **Extension for Nutritional Epidemiology studies (STROBE-nut)** | **Reported on page #** |
| --- | --- | --- | --- | --- |
| Title and  abstract | 1 | (a) Indicate the study’s design with a commonly used term in the title or the abstract.  (b) Provide in the abstract an informative and balanced summary of what was done and what was found. | **nut-1** State the dietary/nutritional assessment method(s) used in the title, abstract, or keywords. | 1, 6 |
| **Introduction** |  |  |  |  |
| Background rationale | 2 | Explain the scientific background and rationale for the investigation being reported. |  | 8-9 |
| Objectives | 3 | State specific objectives, including any pre-specified hypotheses. |  | 9 |
| **Methods** |  |  |  |  |
| Study design | 4 | Present key elements of study design early in the paper. |  | 9 |
| Settings | 5 | Describe the setting, locations, and relevant dates, including periods of recruitment, exposure, follow-up, and data collection. | **nut-5** Describe any characteristics of the study settings that might affect the dietary intake or nutritional status of the participants, if applicable. | 9 |
| Participants | 6 | a) Cohort study—Give the eligibility criteria, and the sources and methods of selection of participants. Describe methods of follow-up.  Case-control study—Give the eligibility criteria, and the sources and methods of case ascertainment and control selection. Give the rationale for the choice of cases and controls.  Cross-sectional study—Give the eligibility criteria, and the sources and methods of selection of participants.  (b) Cohort study—For matched studies, give matching criteria and number of exposed and unexposed.  Case-control study—For matched studies, give matching criteria and the number of controls per case. | **nut-6** Report particular dietary, physiological or nutritional characteristics that were considered when selecting the target population. | 9 |
| Variables | 7 | Clearly define all outcomes, exposures, predictors, potential confounders, and effect modifiers. Give diagnostic criteria, if applicable. | **nut-7.1** Clearly define foods, food groups, nutrients, or other food components.  **nut-7.2** When using dietary patterns or indices, describe the methods to obtain them and their nutritional properties. | 10-13 |
| Data sources - measurements | 8 | For each variable of interest, give sources of data and details of methods of assessment (measurement).Describe comparability of assessment methods if there is more than one group. | **nut-8.1** Describe the dietary assessment method(s), e.g., portion size estimation, number of days and items recorded, how it was developed and administered, and how quality was assured. Report if and how supplement intake was assessed.  **nut-8.2** Describe and justify food composition data used. Explain the procedure to match food composition with consumption data. Describe the use of conversion factors, if applicable.  **nut-8.3** Describe the nutrient requirements, recommendations, or dietary guidelines and the evaluation approach used to compare intake with the dietary reference values, if applicable.  **nut-8.4** When using nutritional biomarkers, additionally use the STROBE Extension for Molecular Epidemiology (STROBE-ME). Report the type of biomarkers used and their usefulness as dietary exposure markers.  **nut-8.5** Describe the assessment of nondietary data (e.g., nutritional status and influencing factors) and timing of the assessment of these variables in relation to dietary assessment.  **nut-8.6** Report on the validity of the dietary or nutritional assessment methods and any internal or external validation used in the study, if applicable. | 10-13 |
| Bias | 9 | Describe any efforts to address potential sources of bias. | **nut-9** Report how bias in dietary or nutritional assessment was addressed, e.g., misreporting, changes in habits as a result of being measured, or data imputation from other sources | 19 |
| Study Size | 10 | Explain how the study size was arrived at. |  | 14 |
| Quantitative variables | 11 | Explain how quantitative variables were handled in the analyses. If applicable, describe which groupings were chosen and why. | **nut-11** Explain categorization of dietary/nutritional data (e.g., use of N-tiles and handling of nonconsumers) and the choice of reference category, if applicable. | 13-14 |
| Statistical  Methods | 12 | (a) Describe all statistical methods, including those used to control for confounding  (b) Describe any methods used to examine subgroups and interactions.  (c) Explain how missing data were addressed.  (d) Cohort study—If applicable, explain how loss to follow-up was addressed.  Case-control study—If applicable, explain how matching of cases and controls was addressed.  Cross-sectional study—If applicable, describe analytical methods taking account of sampling strategy.  (e) Describe any sensitivity analyses. | **nut-12.1** Describe any statistical method used to combine dietary or nutritional data, if applicable.  nut-12.2 Describe and justify the method for energy adjustments, intake modeling, and use of weighting factors, if applicable.  **nut-12.3** Report any adjustments for measurement error, i.e,. from a validity or calibration study. | 13-14 |
| **Results** |  |  |  |  |
| Participants | 13 | (a) Report the numbers of individuals at each stage of the study—e.g., numbers potentially eligible, examined for eligibility, confirmed eligible, included in the study, completing follow-up, and analyzed.  (b) Give reasons for non-participation at each stage.  (c) Consider use of a flow diagram. | **nut-13** Report the number of individuals excluded based on missing, incomplete or implausible dietary/nutritional data. | 14 |
| Descriptive data | 14 | (a) Give characteristics of study participants (e.g., demographic, clinical, social) and information on exposures and potential confounders  (b) Indicate the number of participants with missing data for each variable of interest  (c) Cohort study—Summarize follow-up time (e.g., average and total amount) | **nut-14** Give the distribution of participant characteristics across the exposure variables if applicable. Specify if food consumption of total population or consumers only were used to obtain results. | 14-16 |
| Outcome data | 15 | Cohort study—Report numbers of outcome events or summary measures over time.  Case-control study—Report numbers in each exposure category, or summary measures of exposure.  Cross-sectional study—Report numbers of outcome events or summary measures. |  | 16-17 |
| Main results | 16 | Give unadjusted estimates and, if applicable, confounder-adjusted estimates and their precision (e.g., 95% confidence interval).  Make clear which confounders were adjusted for and why they were included.  (b) Report category boundaries when continuous variables were categorized.  © If relevant, consider translating estimates of relative risk into absolute risk for a meaningful time period. | **Nut-16** Specify if nutrient intakes are reported with or without inclusion of dietary supplement intake, if applicable. | 16-17 |
| Other analyses | 17 | Report other analyses done—e.g., analyses of subgroups and interactions and sensitivity analyses. | **nut-17** Report any sensitivity analysis (e.g., exclusion of misreporters or outliers) and data imputation, if applicable. | 16-17 |
| **Discussion** |  |  |  |  |
| Key results | 18 | Summarize key results with reference to study objectives. |  | 17 |
| Limitation | 19 | Discuss limitations of the study, taking into account sources of potential bias or imprecision. Discuss both direction and magnitude of any potential bias. | **nut-19** Describe the main limitations of the data sources and assessment methods used and implications for the interpretation of the findings. | 19 |
| Interpretation | 20 | Give a cautious overall interpretation of results considering objectives, limitations, multiplicity of analyses, results from similar studies, and other relevant evidence. | **nut-20** Report the nutritional relevance of the findings, given the complexity of diet or nutrition as an exposure. | 17-20 |
| Generalizability | 21 | Discuss the generalizability (external validity) of the study results. |  | 19 |
| **Other information** |  |  |  |  |
| Funding | 22 | Give the source of funding and the role of the funders for the present study and, if applicable, for the original study on which the present article is based. |  | 3-5 |
| Ethics |  |  | **nut-22.1** Describe the procedure for consent and study approval from ethics committee(s). | 3 |
| Supplementary material |  |  | **nut-22.2** Provide data collection tools and data as online material or explain how they can be accessed. | Supplement |

### **Supplementary Table 2.** Classification of discretionary foods and beverages used in the present analysis according to Food Standards Scotland classification

| Discretionary classification | | Food4Me FFQ item |
| --- | --- | --- |
| Food group | Food items included |  |
| Sweet biscuits | Sweet biscuits (not chocolate) and cereal bars, chocolate biscuits, fully-coated chocolate biscuits or wafers, sweet biscuits including half-coated chocolate biscuits, cereal bars and cereal based cakes. | Sweet biscuits, chocolate e.g. digestive, cookies |
|  |  | Sweet biscuits, plain e.g. nice, ginger |
| Cakes, pastries and puddings | Danish pastries, pecan danish, fruit pies, fruit tarts, jam tarts, custard tart, treacle tart, flans, bakewells, chorley cakes, tortes, egg custards, raisin & currant puffs, fruit pastries, sponge cakes, gateau, stollen, parkin, swiss rolls, chocolate cakes, cream cakes, éclairs, chocolate croissant, pain au chocolat, chocolate brioche, meringues, pavlova, pop tarts, doughnuts, american muffin ‘cakes’, blueberry muffins, chocolate muffins, frozen cheesecakes, frozen chocolate filled pancakes, frozen eclairs, frozen sponges and gateaux, (including those with ice-cream), frozen danish, frozen custard slice, frozen apple pie, frozen fruit pies, frozen pavlova, frozen profiteroles. Instant/dessert whips, trifle mixes, cheesecake mixes, crumble mix, fruit puddings, summer fruit pudding, sponge puddings, chocolate sponge pudding, treacle sponge pudding, syrup puddings, fruit fritters, christmas pudding, bread pudding, sticky toffee pudding | Plain cakes e.g. fruit, sponge, scones, gingerbread |
|  |  | Rich cakes e.g. chocolate, cheesecake |
|  |  | Flapjacks, muesli bars, oatmeal cookies |
|  |  | Buns, muffins, pastries e.g. croissants, doughnuts |
|  |  | Waffles, pancakes, crepes |
|  |  | Fruit pies, tarts, crumbles |
|  |  | Sponge puddings |
|  |  | Milk puddings, e.g. rice, custard, trifle |
| Ice cream and dairy desserts | Ice cream tub or block, ice cream cornets, choc-ices, lollies with ice cream, ice lollies, sorbets, chilled dairy desserts including mousse, pannacotta, cheesecake, trifle, syllabub, fruit fool, tiramisu, twin pots, frozen yoghurt, takeaway milkshakes | Ice-cream, choc ices |
|  |  | Sorbets and jellies |
| Confectionery | Solid chocolate bars, filled chocolate-coated bars, sweets, mints, boiled sweets, fudges, toffees, caramels, jellies and unspecified 'sweets,' uncoated toffee or fudge, chocolate éclairs, caramels, pick 'n' mix, nougat, liquorice and other sweets | Chocolates, single or squares |
|  |  | Chocolate snack bars e.g. mars/crunchie |
|  |  | Sweets, toffees, mints, liquorice |
| Crisps and savoury snacks | Crisps and potato snacks, cereal snacks, popcorn, poppadums, prawn crackers, corn snacks (based on maize) and wheat based savoury snacks | Crisps or other packet snacks e.g. wotsits |
| Pastry or snack product | Pastry and snack products | Baklava, kantaifi |
|  |  | Snackbarproducts eg frikandel, kroket, bamibal, kaassouflé |
|  |  | Stroopwaffel |
| Sugar containing soft drinks | Soft drinks, concentrated, not low calorie, soft drinks, not concentrated, not low calorie, soft drink (incl carbonates & still) - not low calorie (including drinks where calorie content unspecified), soft drink where pure juice or juice drink not specified, mixer recorded with spirits and alcopops. | Fizzy soft drinks e.g. coca cola / lemonade |
|  |  | Fruit squash/ cordial / nectar |
|  |  | Sweet alcoholic drinks, e.g. alcopops, cocktails |

Supplementary Table 3. Forty-five food groups used in the creation of the dietary patterns

| Food category | Food group | Food items included |
| --- | --- | --- |
| Cereals  (6 items) | 1. Pasta, rice and cereals | White rice, White Pasta, Noodles And Other Grains E.g. Cous Cous, Polenta, Lasagne, Moussaka, Ravioli And Tortelini, Filled Dumplings |
|  | 1. Whole meal pasta, rice and cereals | Brown Rice, Buckwheat And Barley Groats, Wholemeal Pasta |
|  | 1. White bread | White Rolls, White Bread, Tortillas, Wraps  Cream Crackers, Cheese Biscuits, Rusks  Crispbread E.g. Ryvita, Bread And Savoury Biscuits |
|  | 1. Whole meal bread | Brown Bread And Seeded Bread, Dark Wholemeal Breads E.g. Rye Or Soda, Brown And Seeded Rolls |
|  | 1. High fibre breakfast cereals | Porridge, Readybrek, Breakfast Cereals, Wholegrain E.g. Branflakes, Barley Flakes  Breakfast Cereals E.g. Muesli, Cruesli |
|  | 1. Other breakfast cereals | Breakfast Cereals, Non-Wholegrain E.g. Cornflakes |
| Dairy products  (8 items) | 1. Whole milk | Full Fat Whole Milk Average, Coffee, Milky, Latte, Cappuccino, Milkshakes, Fruit Smoothies |
|  | 1. Skimmed milk | Low-Fat/Semi-Skimmed Milk, Zero Fat/Skimmed Milk |
|  | 1. Cheese | High Fat Cheeses E. g. Stilton, Cheddar, Brie, Gouda  Medium Fat Cheeses E. g. Edam, Goats, Camembert, Feta, Emmental, Low Fat Cheeses - Fresh Mozzarella, Cream Cheese, Katiki |
|  | 1. Low fat cheese | Very Low Fat Cheese e.g. Cottage Cheese, Quark |
|  | 1. Yoghurt low fat | Low-Fat Natural Yoghurt |
|  | 1. Yoghurt full fat | Full Fat Greek Yoghurt, Fruit Yoghurt, Fruit Mousse |
| Fats, spreads and sauces  (3 items) | 1. Butter | Butter |
|  | 1. Margarine and olive oil | Block/Hard Margarine E. g. Stork/Krona, Polyunsaturated Marg Flora/Sunflower/Soya  Soft Marg Olive Oil Based, Bertolli/Blue Band  Low Fat Spreads (< 60%), Olive Oil, Other Vegetable Oils |
|  | 1. Jams, spreads and sauces | Tinned, Stewed Fruit, Jam/Marmalade/Honey  Nut or Chocolate Spreads E. g. Peanut Butter, Nutella, Low Fat Salad Cream, Mayonnaise, Salad Cream, Mayonnaise, Creamy Sauces E. g.carbonara/Cheese, Dark Sauces E.g. Gravy, Stir-Fry Sauces, Tomato Ketchup  Pickles, Chutney, Satesaus (Sate), Marmite/Bovril  French Dressing, Vinaigrette, Other Salad Dressing, Tomato Sauces e. g. In Bolognese, On Meatballs or Pasta, Sugar, Added to Tea, Coffee, Cereal |
| Meat and alternatives  (10 items) | 1. Beef, veal and dishes | Beef, Venison (Roast, Steak, Mince), Lamb, Goat (Roast, Chops, Souvlaki), Stew and Casserole (Meat and Veg), Burgers e. g. Beef, Meatballs |
|  | 1. Crumbed fried poultry | Processed Chicken or Poultry e. g. Nuggets, Goujons, Fried |
|  | 1. Non fried chicken, turkey pork and dishes | Pork (Roast, Chops)  Chicken or Poulty, Grilled, Roast |
|  | 1. Processed meats | Bacon, Sliced Cold Meats E. g. Ham, Turkey  Cured Meats E. g. Corned Beef, Salami, Chorizo  Sausages e. g. Pork, Jadwurst, Savory Pies, Meat Pies, Pasties, Sausage Rolls |
|  | 1. Liver and other meats | Offal E. g. Liver, Kidney, Pate e. g. Meat or Liver |
|  | 1. Battered fish | Fried Fish In Batter, Fish Fingers, Fish Cakes |
|  | 1. Fish and fish dishes | Fish Dishes e.g. Pie, Pudding, Casserole, White Fish E.g. Cod, Haddock, Sole Non-Smoked Oily Fish, Fresh (Mackerel, Tuna, Sardines, Salmon), Non-Smoked Oily Fish,Canned (Mackerel, Tuna, Sardines, Salmon) Smoked Fish E.g. Salmon, Mackerel, Sushi |
|  | 1. Other seafood | Fish Roe, Caviar, Taramasalata, Shellfish, Crab, Prawn |
|  | 1. Meat alternatives | Tofu, Soya Meat, TVP, Vegeburger |
|  | 1. Eggs and eggs dishes | Eggs, Egg - Boiled, Scrambled, Omelette Etc. |
| Fruit and Vegetables  (4 items) | 1. Fruits | Apples, Pears, Oranges, Satsumas, Mandarins  Grapefruit, Bananas, Grapes, Melon, Mango  Peaches/Plums/Apricots, Berries E.g. Strawberries, Raspberries, Cherries, Kiwi, Avocado, Dried Fruit Eg Raisins, Prunes |
|  | 1. Vegetables raw and boiled | Carrots, Butternut Squash,Pumpkin, Spinach, Broccoli, Spring Greens, Kale, Brussel Sprouts  Cabbage, Marrow, Courgettes, Aubergine  Cauliflower, Parsnips, Turnips, Swedes  Asparagus, Okra, Onions, Leeks, Garlic, Mushrooms  Sweet Peppers, Beans Sprouts, Radish  Green Salad, Lettuce, Cucumber, Celery, Watercress, Tomatoes, Corn (On The Cob, Sweetcorn), Beetroot, Coleslaw, Sauerkraut, Olives |
|  | 1. Vegetables (mixed dishes) | Sauerkraut Stew, Stuffed Cabbage  Potato Dishes E.g. Salads, Dauphinoise, Creamy Soups E.g. Chowder, Cream Of Mushroom  Non-Creamy Soups E.g. Minestrone, Vegetable |
|  | 1. Legumes | Baked Beans, Dried Lentils, Beans, Peas, Chickpeas  Fresh/ Frozen Peas, Green Beans, Broad Beans, Runner Beans |
|  | 1. Boiled and baked potato | Potatoes - Mashed, Instant, Roast  Potatoes - Boiled, Jacket |
| Nuts and seeds  (1 item) | 1. Nuts and seeds | Nuts And Seeds E.g. Almonds, Peanuts, Pumpkin Seeds |
| Discretionary snack foods  (7 items) | 1. Sweet biscuits | Sweet Biscuits,Chocolate Eg Digestive, Cookies  Sweet Biscuits, Plain Eg Nice, Ginger |
|  | 1. Cakes, pastries and pudding | Plain Cakes Eg Fruit, Sponge, Scones, Gingerbread, Raisinbread, Rich Cakes E.g. Chocolate, Cheesecake  Flapjacks, Muesli Bars, Oatmeal Cookies  Buns, Muffins, Pastries E.g. Croissants, Doughnuts  Waffles, Pancakes, Crepes, Fruit Pies, Tarts, Crumbles, Sponge Puddings, Milk Puddings, Eg Rice, Custard, Trifle, Baklava, Kantaifi, Snackbarproducts Eg Frikandel, Kroket, Bamibal, Kaassoufl, Stroopwafle, Quiche, Savoury Pancakes |
|  | 1. Ice cream and dairy dessert | Ice-Cream, Choc Ices, Sorbets And Jellies, Single/Sour Cream, Double/Clotted Cream |
|  | 1. Confectionary | Chocolates, Single Or Squares, Chocolate Snack Bars Eg Mars/Crunchie, Sweets, Toffees, Mints, Liquorice |
|  | 1. Crisp and savory snacks | Crisps Or Other Packet Snacks Eg Wotsits |
|  | 1. Pizza or snack products^1^ | Pizza, Calzone, Springrolls, Potato Or Plain Dumplings and chips |
|  | 1. Sugar containing soft drinks | Sweet Alcoholic Drinks, Eg Alcopops, Cocktails  Fizzy Soft Drinks E.g. Coca Cola / Lemonade  Fruit Squash/ Cordial / Nectar |
| Non-alcoholic beverages  (4 items) | 1. Tea and coffee | Tea (Black, Green, Fruit, Herbal), Coffee, Americano, Black |
|  | 1. High sugar beverage | Hot Chocolate, Ovaltine, Horlicks Made With Milk  Hot Chocolate, Ovaltine, Horlicks, Made With Water |
|  | 1. Low calorie drink | Low Calorie/ Diet Fizzy Soft Drinks |
|  | 1. Fruit and vegetable juice | Pure Fruit Juice E.g. Orange, Tomato And Vegetable Juices |
| Alcoholic beverages  (3 items) | 1. Wine | Wine |
|  | 1. Beer and cider | Beer, Larger, Cider |
|  | 1. Spirits and other alcohol | Port, Sherry, Vermouth, Liqueurs  Spirits, E.g. Gin, Brandy, Whiskey, Vodka |
| 1, Pizza and snack products were not included in the calculation of %E intake from discretionary food | | |

Supplementary Table 4. Participant characteristics (n = 1,280)

| Characteristics | All  (n= 1 280) | Underweight/  normal weight  (n = 692) | Overweight/  obesity  (n = 588) | P value^1^ |
| --- | --- | --- | --- | --- |
| Age, years (mean, SD) | 40.4 | 37.3 | 44 | <0.001 |
| Female (%) | 58.1 | 63.9 | 51.2 | <0.001 |
| Country (%) |  |  |  | <0.001 |
| Germany | 13.6 | 16.2 | 10.5 |  |
| Greece | 13.6 | 10.3 | 17.5 |  |
| Ireland | 14.0 | 13.4 | 14.6 |  |
| Netherlands | 16.7 | 18.9 | 14.1 |  |
| Poland | 13.9 | 15.0 | 12.4 |  |
| Spain | 14.1 | 11.2 | 17.7 |  |
| United Kingdom | 14.1 | 15.0 | 13.2 |  |
| Smoker (%) | 11.4 | 8.8 | 14.5 | 0.002 |
| Physical activity (%) |  |  |  | <0.001 |
| Do not meet guidelines | 22.7 | 12.0 | 35.2 |  |
| Meet guidelines | 77.3 | 88.0 | 64.8 |  |
| *FTO rs99397609* (%) |  |  |  | 0.02 |
| TT | 31.6 | 35.1 | 27.6 |  |
| TA | 50.2 | 45.0 | 51.5 |  |
| AA (risk variant) | 18.2 | 15.9 | 20.9 |  |

SD, standard deviation; Underweight/normal weight: body mass index <25 kg/m^2^, Overweight/obesity: body mass index ≥25 kg/m^2^

^1^P value for unadjusted linear regression analyses of the association between participant characteristics across participants with underweight/normal weight and overweight/obesity.

Supplementary Table 5. Energy and nutrient intake across tertiles of dietary patterns (n = 1,280)

|  | Dietary pattern | | | | | | - 1. P_trend_^1^ |
| --- | --- | --- | --- | --- | --- | --- | --- |
|  | Tertile 1 | | Tertile 2 | | Tertile 3 | |  |
|  | Mean | SD | Mean | SD | Mean | SD |  |
| Dietary pattern 1 |  |  |  |  |  |  |  |
| Total energy (MJ/day) | 10.5 | 3.83 | 9.48 | 3.44 | 12.2 | 5.41 | <0.001 |
| Carbohydrate (%E/day) | 49.2 | 7.6 | 45.0 | 6.8 | 43.8 | 7.3 | <0.001 |
| Total sugar (%E/day) | 22.3 | 6.7 | 20.3 | 5.0 | 20.5 | 5.6 | <0.001 |
| Fibre (g/day) | 38.1 | 15.2 | 25.6 | 10.5 | 25.5 | 12.5 | <0.001 |
| Protein (%E/day) | 16.9 | 3.5 | 17.6 | 3.6 | 16.6 | 2.7 | 0.27 |
| Total fat (%E/day) | 32.5 | 5.6 | 35.9 | 4.9 | 39.4 | 5.0 | <0.001 |
| Polyunsaturated fat (%E/day) | 5.83 | 1.61 | 5.71 | 1.44 | 5.72 | 1.32 | 0.28 |
| Omega-3 (g/day) | 2.09 | 1.15 | 1.83 | 0.81 | 2.11 | 1.10 | 0.83 |
| Monounsaturated fat (%E/day) | 12.6 | 3.2 | 13.8 | 2.9 | 14.7 | 2.8 | <0.001 |
| Trans fat (%E/day) | 0.36 | 0.13 | 0.48 | 0.15 | 0.56 | 0.17 | <0.001 |
| Alcohol (%E/day) | 3.52 | 4.33 | 3.63 | 3.53 | 2.74 | 3.00 | 0.002 |
| Dietary pattern 2 |  |  |  |  |  |  |  |
| Total energy (MJ/day) | 10.9 | 4.10 | 9.46 | 3.52 | 11.8 | 5.27 | 0.008 |
| Carbohydrate (%E/day) | 41.3 | 7.1 | 46.2 | 6.4 | 50.5 | 6.2 | <0.001 |
| Total sugar (%E/day) | 18.3 | 5.4 | 21.4 | 5.3 | 23.4 | 5.8 | <0.001 |
| Fibre (g/day) | 25.9 | 12.0 | 27.0 | 11.9 | 36.3 | 15.9 | <0.001 |
| Protein (%E/day) | 18.4 | 4.1 | 17.3 | 3.4 | 15.4 | 2.9 | <0.001 |
| Total fat (%E/day) | 38.4 | 5.9 | 35.4 | 5.1 | 34.0 | 5.8 | <0.001 |
| Polyunsaturated fat (%E/day) | 5.93 | 1.53 | 5.75 | 1.48 | 5.60 | 1.38 | 0.001 |
| Omega-3 (g/day) | 2.08 | 1.02 | 1.84 | 1.04 | 2.12 | 1.04 | 0.69 |
| Monounsaturated fat (%E/day) | 14.5 | 3.3 | 13.7 | 2.9 | 12.9 | 2.9 | <0.001 |
| Trans fat (%E/day) | 0.53 | 0.18 | 0.46 | 0.16 | 0.41 | 0.15 | <0.001 |
| Alcohol (%E/day) | 4.01 | 4.26 | 3.38 | 3.58 | 2.50 | 2.91 | <0.001 |
| Dietary pattern 3 |  |  |  |  |  |  |  |
| Total energy (MJ/day) | 10.3 | 4.88 | 9.49 | 3.63 | 11.4 | 4.50 | 0.69 |
| Carbohydrate (%E/day) | 45.7 | 7.9 | 46.1 | 7.1 | 46.3 | 7.6 | 0.22 |
| Total sugar (%E/day) | 20.0 | 6.0 | 21.1 | 5.5 | 22.0 | 6.1 | <0.001 |
| Fibre (g/day) | 26.3 | 13.1 | 27.0 | 11.5 | 35.9 | 15.6 | <0.001 |
| Protein (%E/day) | 17.8 | 4.1 | 17.2 | 3.4 | 16.2 | 3.4 | <0.001 |
| Total fat (%E/day) | 33.8 | 5.3 | 35.9 | 5.5 | 38.0 | 6.1 | <0.001 |
| Polyunsaturated fat (%E/day) | 5.34 | 1.31 | 5.82 | 1.41 | 6.11 | 1.56 | <0.001 |
| Omega-3 (g/day) | 2.08 | 0.97 | 1.80 | 0.79 | 2.16 | 1.27 | 0.24 |
| Monounsaturated fat (%E/day) | 13.3 | 3.0 | 13.9 | 3.2 | 13.9 | 3.2 | 0.007 |
| Trans fat (%E/day) | 0.45 | 0.17 | 0.46 | 0.16 | 0.50 | 0.19 | <0.001 |
| Alcohol (%E/day) | 4.97 | 4.70 | 3.01 | 3.03 | 1.92 | 2.15 | <0.001 |
| %E, percentage from total energy intake, kJ, kilojoules, g, grams.  ^1^P for trend from unadjusted linear regression analysis across tertiles of dietary pattern. | | | | | | | |

**Supplementary Table 6.** Factor loadings of the 45 food groups for dietary patterns 1, 2 and 3.

| Food groups | Factor loadings | | |
| --- | --- | --- | --- |
|  | Dietary pattern 1 | Dietary pattern 2 | Dietary pattern 3 |
| Pasta, rice and cereals | 0.11 | -0.11 | -0.08 |
| Whole meal pasta, rice and cereals | **-0.17** | 0.07 | 0.08 |
| White bread | -0.04 | 0.05 | **-0.21** |
| Whole meal bread | **-0.21** | 0.09 | 0.14 |
| High fibre breakfast cereals | **-0.16** | 0.11 | 0.14 |
| Other breakfast cereals | 0.07 | 0.06 | **0.14** |
| Whole milk | **0.20** | **-0.23** | **0.16** |
| Skimmed milk | 0.01 | -0.06 | -0.13 |
| Cheese | 0.14 | **-0.20** | **0.23** |
| Low fat cheese | -0.03 | -0.09 | 0.11 |
| Yoghurt low fat | -0.12 | -0.00 | 0.07 |
| Yoghurt full fat | 0.01 | -0.05 | 0.01 |
| Butter | **0.25** | **-0.18** | **0.39** |
| Margarine and olive oil | -0.01 | -0.09 | -0.10 |
| Jams, spreads and sauces | **0.15** | 0.00 | 0.00 |
| Beef, veal and dishes | 0.10 | **-0.16** | -0.05 |
| Crumbed fried poultry | 0.11 | 0.02 | -0.09 |
| Non-fried chicken, turkey pork and dishes | 0.12 | **-0.16** | **-0.25** |
| Processed meats | **0.18** | -0.14 | -0.08 |
| Liver and other meats | 0.06 | -0.12 | -0.07 |
| Battered fish | 0.09 | -0.02 | -0.12 |
| Fish and fish dishes | -0.07 | -0.05 | -0.12 |
| Other seafood | -0.02 | -0.02 | -0.02 |
| Meat alternatives | -0.13 | 0.12 | 0.13 |
| Eggs and eggs dishes | 0.06 | **-0.18** | 0.02 |
| Fruits | **-0.31** | **0.25** | **0.29** |
| Vegetables raw and boiled | **0.23** | 0.11 | **0.30** |
| Vegetables (mixed dishes) | -0.02 | 0.05 | 0.08 |
| Legumes | **-0.15** | **0.17** | 0.13 |
| Boiled and baked potato | 0.06 | 0.06 | -0.00 |
| Nuts and seeds | -0.12 | 0.11 | 0.16 |
| Sweet biscuits | **0.34** | **0.47** | 0.05 |
| Cakes, pastries and pudding | **0.25** | **0.29** | -0.02 |
| Ice cream and dairy dessert | **0.15** | 0.09 | 0.08 |
| Confectionary | **0.33** | **0.39** | 0.11 |
| Crisp and savory snacks | **0.17** | **0.17** | -0.05 |
| Pizza or snack products | **0.21** | -0.05 | -0.09 |
| Sugar containing soft drinks | 0.13 | **0.15** | **-0.20** |
| Tea and coffee | -0.05 | 0.11 | **0.17** |
| High sugar beverage | 0.02 | -0.00 | -0.09 |
| Low calorie drink | 0.06 | 0.09 | -0.13 |
| Fruit and vegetable juice | -0.06 | 0.04 | -0.12 |
| Wine | -0.06 | -0.05 | -0.13 |
| Beer and cider | 0.02 | -0.10 | **-0.28** |
| Spirits and other alcohol | -0.00 | -0.00 | **-0.15** |
| *Factor loadings higher or equal to 0.15 are bold. Negative factor loading have grey shading. | | | |

Supplementary Table 7. Associations between dietary patterns and anthropometrics, with interaction effects by *FTO* genotype (n = 1 280)

|  | Beta coefficient | 95% CI | P value^1^ | P interaction^2^ |
| --- | --- | --- | --- | --- |
| Body Mass Index (kg/m^2^) |  |  |  |  |
| Dietary pattern 1 | 0.64 | 0.44, 0.84 | <0.001 | 0.15 |
| Dietary pattern 2 | -0.21 | -0.46, 0.05 | 0.11 | 0.97 |
| Dietary pattern 3 | -0.28 | -0.53, 0.02 | 0.037 | 0.81 |
| Waist circumference (cm) |  |  |  |  |
| Dietary pattern 1 | 1.58 | 1.08, 2.07 | <0.001 | 0.05 |
| Dietary pattern 2 | -0.33 | -0.96, 0.29 | 0.30 | 0.51 |
| Dietary pattern 3 | -0.67 | -1.32, -0.03 | 0.039 | 0.31 |
| ^1^P value from linear regression analysis adjusted for age, sex, smoking status, country and physical activity.  ^2^P for interaction between dietary pattern (continuous) and *FTO* risk genotype (categorical) on anthropometrics (continuous). Linear regression analysis adjusted for age, sex, smoking status, country and physical activity. | | | | |

Supplementary Table 8. Associations between dietary patterns and anthropometrics, with interaction effects by *FTO* genotype after excluding energy misreporters (n = 1 018)

|  | Beta coefficient | 95% CI | P value^1^ | P interaction^2^ |
| --- | --- | --- | --- | --- |
| Body Mass Index (kg/m^2^) |  |  |  |  |
| Dietary pattern 1 | 0.69 | 0.28, 1.12 | <0.001 | 0.43 |
| Dietary pattern 2 | -0.41 | -0.94, 0.12 | 0.13 | 0.92 |
| Dietary pattern 3 | -0.44 | -0.90, 0.02 | 0.06 | 0.92 |
| Waist circumference (cm) |  |  |  |  |
| Dietary pattern 1 | 2.06 | 1.03, 3.09 | <0.001 | 0.26 |
| Dietary pattern 2 | -1.45 | -2.78, 0.14 | 0.030 | 0.21 |
| Dietary pattern 3 | -1.69 | -2.83, -0.55 | 0.004 | 0.26 |
| ^1^P value from linear regression analysis adjusted for age, sex, smoking status, country, physical activity and energy misreporting.  ^2^P for interaction between dietary pattern (continuous) and *FTO* risk genotype (categorical) on anthropometrics (continuous). Linear regression analysis adjusted for age, sex, smoking status, country, physical activity and energy misreporting. | | | | |
